# Multi‐site performance of the telemedicine retinopathy of prematurity severity score (tROP‐SS)

**DOI:** 10.1111/aos.70101

**Published:** 2026-02-23

**Authors:** Sarthak V. Shah, Arthur R. Brant, Cindy Zhao, Edward H. Wood, Shannon D. Scarboro, Jochen Kumm, Darius M. Moshfeghi

**Affiliations:** ^1^ Department of Ophthalmology, Horngren Family Vitreoretinal Center, Byers Eye Institute, Stanford University School of Medicine Palo Alto California USA; ^2^ Austin Retina Associates Austin Texas USA; ^3^ Department of Ophthalmology, Dell Medical School University of Texas Austin Austin Texas USA

**Keywords:** modified ROP activity score, retinopathy of prematurity, telemedicine, telemedicine ROP severity score

## Abstract

**Objective:**

This study aims to evaluate the performance of the telemedicine retinopathy of prematurity severity score (tROP‐SS) across all 14 NICUs in the TELEROP cohort against the modified ROP activity score (mROP‐ActS).

**Design:**

Retrospective cohort study.

**Subjects:**

The study included 2037 neonates who underwent ROP screening in 14 NICUs via the TELEROP telemedicine network from November 2017 to January 2025.

**Methods:**

We compared the robustness of tROP‐SS and mROP‐Act systems across all exams.

**Main Outcome Measures:**

The primary outcomes studied were the ability to return a score in cases responding to treatment, correlation between the two scores and the ability to assess disease directionality.

**Results:**

The study analysed 192 704 photos from 11 368 ROP exams on 2037 patients. tROP‐SS generated scores for 100% of exams, demonstrating significant improvements over mROP‐ActS, which scored only 92.3% of all exams (*p* = 0.0136). All patients who met treatment criteria received treatment in both eyes. Among high‐risk and treatment warranted (TW) patients, 100.0% exams had a valid tROP‐SS exam compared with only 72.73% and 45.27% with a valid mROP‐ActS. Only 20.9% high‐risk or TW patients had a valid mROP‐ActS across all their exams. Incremental increases in tROP‐SS were more strongly related to worsening of disease: OR (95% CI) – 3.37 (2.51–4.53) versus 1.57 (1.46–1.69), both *p* < 0.0001. tROP‐SS also demonstrated improved granularity and accuracy in categorizing patients that would ultimately require treatment (AUC: 0.991 vs. 0.825).

**Conclusions:**

tROP‐SS was retrospectively evaluated across 14 NICUs, proving to be a more robust scoring system compared to mROP‐ActS. The findings support continued use of tROP‐SS, supplemented by standard screening protocols to ensure no cases of treatment‐warranted ROP are missed. Future research may focus on integrating additional parameters to enhance predictive models to improve early ROP detection.

## 1 INTRODUCTION

Despite significant advances in neonatal care and the understanding of retinopathy of prematurity (ROP) pathogenesis, ROP remains a leading cause of childhood blindness worldwide (Lim et al., 2023). Universal screening of infants at risk for ROP combined with early detection and timely treatment are critical in preventing visual impairment and blindness (Al‐Abaiji et al., 2025; Hong et al., 2022). Telemedicine‐based ROP screening has been validated and, after maturation over the past two decades, is now performed across numerous neonatology intensive care units (NICU) in the United States with 100% sensitivity for treatment‐warranted disease with no retinal detachments from screening failures (Barrero‐Castillero et al., 2020; Smith et al., 2022; Vartanian et al., 2017). To communicate effectively with neonatologists and family, an easily interpretable scoring system that demonstrates disease severity and trajectory is essential.

We introduced the Telemedicine ROP Severity Score (tROP‐SS) to build upon the work of the validated modified ROP Activity Score (mROP‐ActS) (Pivodic et al., 2021) to function as a dedicated telemedicine‐based scoring system that automatically returns a single value incorporating the metrics of Zone, Stage and Plus using International Classification of Retinopathy of Prematurity, Third Edition (ICROP3) terminology (Chiang et al., 2021; Xu et al., 2023). A retrospective study from a single site over a 9‐year period favoured the use of tROP‐SS over the mROP‐ActS due to a higher percentage of valid scores and increased accuracy in reflecting subsequent treatment need (Xu et al., 2023). Until now, the tROP‐SS has not been evaluated in a multi‐site clinical cohort, and further longitudinal data are necessary for validation and generalizability.

The primary aim of the current study was to evaluate the performance of the tROP‐SS using data from all infants screened for ROP over a 7‐year period in the combined Stanford‐Pediatrix TELEROP screening programme, an administrative subdivision of the overall Stanford telemedicine for ROP programme. The TELEROP screening programme contains a diverse cohort of patients spanning 14 neonatal intensive care units in Arizona, Georgia, Nevada, North Carolina and Texas (Pasricha et al., 2021). We wished to investigate the ability to return a score in high risk and treatment‐warranted cohorts, as well as the correlation between the two scores. As a secondary outcome, we assessed longitudinal trends of disease progression in relation to postmenstrual age and the ability of both scoring systems to accurately assess the directionality of disease severity.

## 2 METHODS

All patients who underwent telemedicine ROP screening in the TELEROP network between November 2017 and January 2025 were included in this retrospective cohort study. Summary statistics were abstracted for the population, including estimated gestational age (EGA), birth weight (BW) and maximum severity of retinopathy of prematurity. All images were taken with the 130° wide‐angle imaging systems coupled with gel to the cornea (PanoCam Solo, Visunex Medical Systems Co. Ltd. and RetCam, Natus Medical Inc.) (Wood et al., 2016). The images were taken by a trained nurse in the neonatal intensive care unit and transferred using a Health Insurance Portability and Accountability Act‐compliant picture archiving and communication system for interpretation. Retinopathy of prematurity (ROP) stage, zone and status of plus disease were defined according to ICROP3 (Chiang et al., 2021). Based on zone, stage and plus classifications, each eye at each visit was automatically scored for both the tROP‐SS and the mROP‐ActS based on previously published tabular scoring. Graders assessing images for clinical management did not have access to conversion tables to either scoring system while grading.

Recently, the terms micro‐ and nano‐premature have been promulgated to describe subsets of ROP‐screening eligible infants with extremely low gestational age and very low birthweights that are at increased risk for needing intervention (Scarboro et al., 2024). We have included these descriptions in our analyses of both scoring systems.

This study was conducted in strict accordance with the ethical standards of the Declaration of Helsinki, and the institutional review board approved all research protocols (IRB #8752). Statistical analyses and plots were generated with Python version 3.11.8 software (Python Software Foundation) and R version 4.4.3.

### 2.1 Descriptive analysis

We calculated the percentage of exams per eye for which tROP‐SS and mROP‐ActS were able to be generated for the following outcomes: (1) overall, (2) low risk (maximum tROP‐SS < 26), (3) moderate risk (maximum tROP‐SS ≥26 and < 40), (4) high risk (maximum tROP‐SS ≥40 and < 55), (5) TW‐ROP defined by Type 1 disease or aggressive retinopathy of prematurity (AROP) and (6) treated patients who included both treatment‐warranted (TW) and referral‐warranted (RW) disease that received off‐label intravitreal bevacizumab or laser.

The RW patients who received treatment prior to a score of 55 occurred because of the following reasons: treatment physician decision to evaluate while performing a treatment on another infant, transport from one NICU to the treating NICU resulted in conversion from RW to TW, documented by the treater (but without corresponding image documentation) and treater availability resulted in treatment before TW (e.g. treatment physician would be unavailable and after a discussion with the family, agreed to treat prior to TW level). Reasons for inability to calculate scores were summarized.

### 2.2 Scoring system correlation

Logistic regression analysis with calculated odds ratios and 95% confidence intervals was performed for each scoring system tROP and mROP‐ActS independently to assess their association with treatment status with incremental score increased by 5% to normalize for differing scoring scales. The spearman rank correlation coefficient was used to assess correlation between the tROP‐SS and mROP‐ActS per each eye.

### 2.3 Assessment of classification accuracy

Using real‐world treatment data, receiver operating curves were made and area under the curve was calculated to compare classification accuracy by tROP‐SS and mROP‐ActS.

### 2.4 Disease progression analysis

Among patients with valid maximum tROP‐SS and mROP‐ActS, disease progression from 30 weeks to 40 weeks post menstrual age (PMA) was assessed with a 95% confidence interval based on the worse eye at each visit. Subgroup analysis was done to compare disease progression between treated and untreated patients, and separately between nano, micro and not nano/micro classifications by EGA and BW. The plots of disease progression over time (i.e. ‘tempo’) was graphed for both the tROP‐SS and mROP‐ActS and evaluated relative to PMA. To assess scores progression between 29‐ and 35‐week PMA, we employed a Bayesian linear mixed‐effects model. Separate models were fitted for each combination of scoring system (tROP‐SS and mROP‐ActS) and treatment status (treated and untreated). We accounted for a population‐level fixed effect of PMA on the ROP score as well as patient‐specific random effects, accounting for individual variations in both baseline scores (intercepts) and rates of change (slopes). The models were fitted using Markov Chain Monte Carlo (MCMC) sampling with four chains, each running for 2000 iterations with a warm‐up period of 1000 iterations. To compare the slopes between different models (e.g. treated vs. untreated, tROP‐SS vs. mROP‐ActS), we extracted the posterior distributions of the fixed effect slopes from each model and then calculated the mean difference in slopes with a 95% CI as well as the probability of direction (Figure S1).

## 3 RESULTS

The study analysed 192 704 photos from 11 368 ROP exams on 2037 unique patients who were abstracted from 7 years of continuous data from 14 NICUs in the TELEROP network. 22 736 eye scores were generated for each scoring system. Demographic data are summarized in Table 1. The 49 treated patients were inclusive of both TW and RW groups and received intravitreal bevacizumab in both eyes. RW patients who received treatment despite having imaging findings below the level of Type I ROP occurred due to an in‐person treater's assessment as opposed to the telemedicine grader assessment. High‐risk, TW and treated patients had the highest numbers of invalid mROP‐ActS: 27.27%, 54.73% and 59.12%, respectively. Only 20.9% high‐risk or TW patients had a valid mROP‐ActS across all their exams. For all visits per eye with invalid mROP‐ActS, 59.53% were explained by only ‘Posterior Zone II’, 24.13% by only ‘Regression after treatment’ and 12.92% by only ‘pre‐plus disease’ (Table S1). Less common reasons included some combination of these three factors as well as ‘Reactivation after treatment’ (0.11%) and ‘Zone I, Incomplete Vascularization with Plus’ (0.17%).

**TABLE 1:** Clinical characteristics of TELEROP cohort.

	All patients	Low risk^a^	Moderate risk^b^	High Risk^c^	TW‐ROP^d^	Treated patients
N (patients)	2037	1701	269	34	33	49
EGA^e^ (std in days)	29/2 (24)	29/2 (22)	25/3 (12)	24/2 (11)	23/2 (9)	23/2 (9)
Birth weight in grams (std)	1267 (573)	1375 (562)	745 (179)	632 (153)	592 (128)	606 (138)
Category						
Nano combined^f^	54 (2.7%)	5 (0.3%)	26 (9.7%)	10 (29.4%)	13 (39.4%)	18 (36.7%)
Nano birth weight^g^	65 (3.2%)	22 (1.3%)	31 (11.5%)	7 (20.6%)	5 (15.2%)	8 (16.3%)
Nano EGA^h^	30 (1.5%)	6 (0.4%)	14 (5.2%)	4 (11.8%)	6 (18.2%)	7 (14.3%)
Micro combined^i^	106 (5.2%)	37 (2.2%)	57 (21.2%)	6 (17.6%)	6 (18.2%)	10 (20.4%)
Micro birth weight^j^	104 (5.1%)	53 (3.1%)	49 (18.2%)	1 (2.9%)	1 (3.0%)	1 (2.0%)
Micro EGA^k^	67 (3.3%)	39 (2.3%)	23 (8.6%)	4 (11.8%)	1 (3.0%)	4 (8.2%)
Non‐micro Non‐nano^l^	1611 (79.1%)	1539 (90.5%)	69 (25.7%)	2 (5.9%)	1 (3.0%)	1 (2.0%)
Sex						
Male	1065 (52.3%)	893 (52.5%)	134 (49.8%)	19 (55.9%)	19 (57.6%)	29 (59.2%)
Female	971 (47.7%)	807 (47.4%)	135 (50.2%)	15 (44.1%)	14 (42.4%)	20 (40.8%)
Average max tROP‐SS (std)	19.00 (9.02)	15.74 (2.09)	30.59 (1.52)	42.29 (3.33)	68.24 (12.18)	58.76 (17.49)
Average max mROP‐ActS (std)	1.07 (3.04)	0.37 (1.42)	2.78 (3.87)	10.48 (2.74)	14.94 (4.38)	12.50 (6.01)
% Visits with invalid tROP‐SS	0.00%	0.00%	0.00%	0.00%	0.00%	0.00%
% Visits with invalid mROP‐ActS	7.73%	3.58%	11.71%	27.27%	54.73%	59.12%

^a^ Low risk is defined as having a maximum tROP‐SS in either eye of less than 26.

^b^ Moderate risk is defined having a maximum tROP‐SS in either eye greater than or equal to 26 and less than 40.

^c^ High risk is defined having a maximum tROP‐SS in either eye greater than or equal to 40 and less than 55.

^d^ TW: Treatment warranted is defined as having a maximum tROP‐SS in either eye of greater than or equal to 55.

^e^ EGA: Estimated gestational age.

^f^ Nano combined is defined as having both nano birth weight and nano EGA.

^g^ Nano birth weight as having a birth weight of less than or equal to 600.

^h^ Nano EGA is defined as having an EGA less than or equal to 24 weeks.

^i^ Micro combined is defined as having both micro birth weight and micro EGA.

^j^ Micro birth weight as having a birth weight of greater than 600 and less than 800.

^k^ Micro EGA is defined as having an EGA greater than 24 weeks and less than or equal to 26 weeks.

^l^ Non‐nano and non‐micro are defined as having a birth weight of greater than or equal to 800 and an EGA of greater than 26 weeks.

**FIGURE 1:**
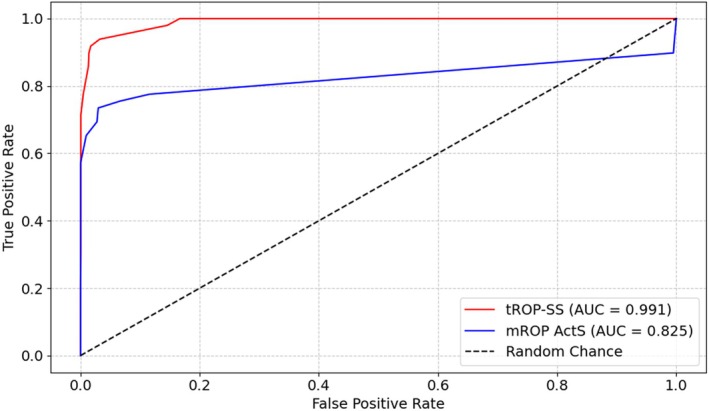
Receiver operating characteristic curves for tROP‐SS and mROP‐ActS.

Independent logistic regressions of each scoring system showed that incremental increases in tROP‐SS were more strongly related to worsening of disease: OR (95% CI) – 3.37 (2.51–4.53) versus 1.57 (1.46–1.69), both *p* < 0.0001. Overall correlation of the tROP‐SS to the mROP‐ActS was strong, *r* = 0.71 for the left eye and *r* = 0.67 for the right eye, *p* < 0.0001. Of the 33 patients who had Type 1 ROP, 100% had tROP‐SS scores greater than or equal to the defined threshold of 55 compared to 84.8% that had mROP‐ActS scores greater than or equal to the threshold of 13. Of the total patients treated, 16 had RW disease, all with tROP‐SS scores below the defined threshold of 55, ranging from 25 to 53. ROC curves made based on real‐world treatment data (consisting of TW‐ROP and RW‐ROP) showed tROP‐SS with a superior AUC compared to the mROP‐ActS – AUC: 0.991 vs. 0.825 (Figure 1).

**FIGURE 2:**
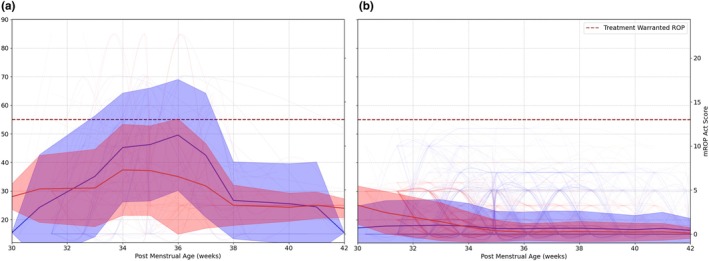
Treated (a) and untreated (b) distributions of geometrically scaled scores with shaded regions depicting a 95% confidence band, and the dashed line indicating the score defining Treatment Warranted ROP for both tROP‐SS (red) and mROP‐ActS (blue).

**FIGURE 3:**
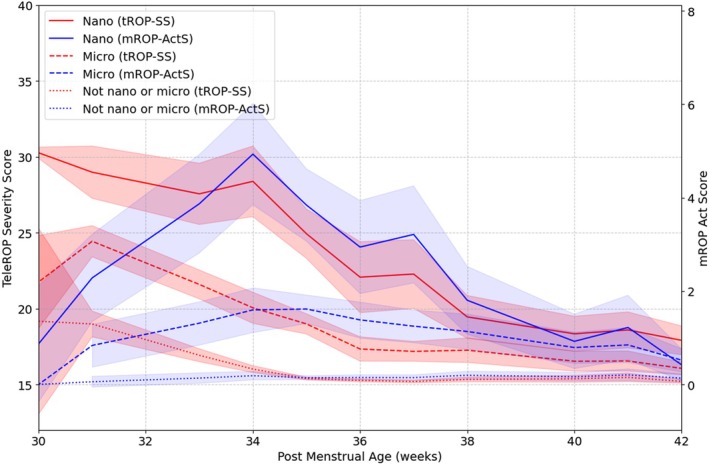
Distribution of tROP‐SS and mROP‐ActS over postmenstrual age in nano and micro patients. Nano is defined as having a birth weight of less than or equal to 600 grams or an estimated gestational age less than or equal to 24 weeks. Micro is defined as having a birth weight greater than 600 grams and less than 800 grams or an estimated gestational age greater than 24 weeks and less than or equal to 26 weeks.

On comparison of disease progression over PMA, tROP‐SS consistently showed earlier peaking of scores than the mROP‐ActS (Figures 2 and 3). Due to fewer valid scores, mROP‐ActS consistently had wider distributions and variability across all PMAs. Both scoring systems peaked at 36‐week PMA in the treated cohort, although there was significant variability in the PMA at which criteria for TW‐ROP were met (Figure 2). Nano babies had consistently higher scores across all PMAs up to 38 weeks, peaking at 34 weeks PMA for the mROP‐ActS and 30 weeks for the tROP‐SS (Figure 3).

**FIGURE 4:**
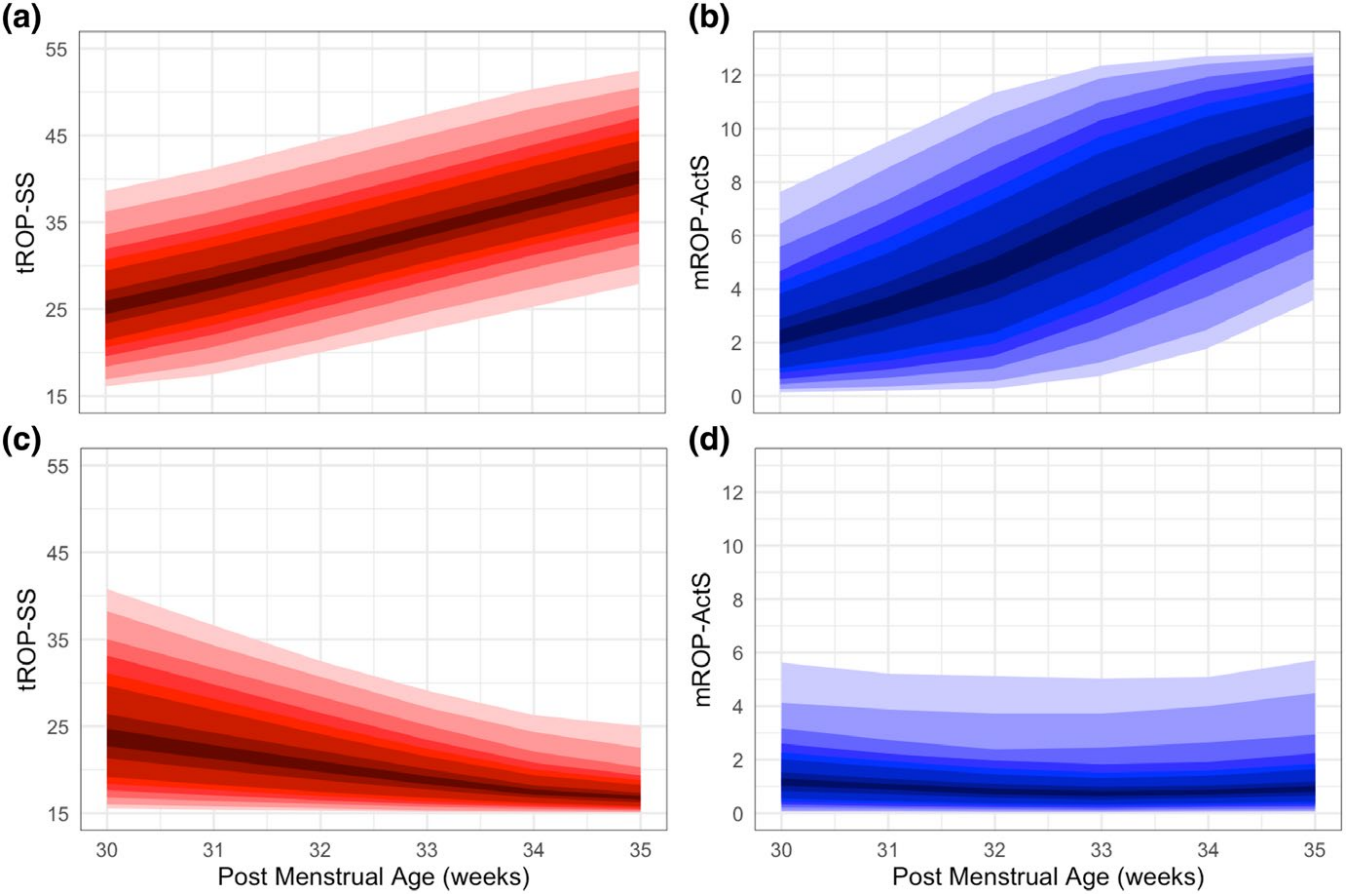
Treated (a, b) and untreated (c, d) population plot with 95% confidence intervals created with posterior predicted means. This type of prediction incorporates the uncertainty for the population average (i.e. the fixed effects) and the population variation (i.e. the random effects). tROP‐SS (a, c) distributions are displayed in red, and mROP‐ActS (b,d) distributions are displayed in blue.

Between 29‐ and 35‐week PMA, the treated subgroups of tROP‐SS and mROP‐ActS had significantly steeper slopes than their untreated counterparts (mean difference: 4.53, 95% CI: 2.74, 6.30, PD: 100% and mean difference: 2.79, 95% CI: 1.98, 3.51, PD: 100%, respectively). mROP‐ActS had consistently wider distributions than tROP‐SS due to fewer valid scores (Figure 4). Slopes were not significantly different between the treated subgroups of both scoring systems (mean difference: 0.33, 95% CI: −1.61, 2.30, PD: 63.2%). Among the untreated subgroup, tROP‐SS notably had a negative slope compared to mROP‐ActS with a positive slope (mean difference: −1.42, 95% CI: −1.58, −1.26, PD: 0%).

## 4 DISCUSSION

The growing adoption of telemedicine‐based ROP screening has highlighted the importance of a scoring system that is both straightforward for ophthalmologists, paediatricians and families but also provides a nuanced representation of disease severity. The tROP‐SS was developed to create severity scores that account for all exam findings in the ICROP3 and provide sufficient granularity to highlight smaller changes in exam (Xu et al., 2023). Initially evaluated retrospectively in a single NICU, we present a more robust comparison of tROP‐SS against mROP‐ActS in all 14 NICUs affiliated with the TELEROP network.

We found a strong correlation between scoring systems but notable differences in the ability to return valid scores, especially among TW and high‐risk patients. Among patients screened for ROP in our data set, 2.4% of patients required treatment. It is unknown at this time why the intervention rate is relatively low and could be attributable to population variability or benefits of longitudinal interval comparison resulting in less need for intervention. tROP‐SS was able to generate scores for 100% of patient exams, compared with mROP‐ActS scores for 92.3% of all exams and 40.04% among treated patients. Notably, tROP‐SS had a higher AUC displaying increased classifier accuracy with real‐world treatment data. The tROP‐SS is also notably more granular with a higher predictive probability for requiring treatment (3X vs. 1.6X OR) at 5% incremental increases in scores.

In relation to disease progression over PMA, the tROP‐SS consistently flagged risk earlier with peaking scores before the mROP‐ActS in all sub‐groups (TW, Nano, Micro, not Nano or Micro). This can be explained because the tROP‐SS unbundles zone, stage and plus, with higher relative weights on Zone I, Stage 3 and Plus. An infant with Zone I, Incomplete, No Plus is flagged as a higher risk infant in tROP‐SS by virtue of having Zone I, whereas in the mROP‐ActS, the lack of Stage and lack of Plus confer a lower risk score on the infant. The mROP‐ActS is discounting the Zone I risk, which can have screening implications and vision implications. The tROP‐SS is specifically designed for use in telemedicine because of how telemedicine employs follow‐up–mandated screening interval of weekly or more often–as opposed to in‐person binocular indirect ophthalmoscopy which employs a findings‐based screening interval. The mROP‐ActS is designed for categorizing all the disease in an eye, as opposed to assessing risk for TW‐ROP interventions. When graphically plotted over time, the tROP score (Figure 2) places the risk of Zone I high, whereas the mROP‐ActS plots it low (discounting), but if either Stage or Plus develop, it appears to catch up. This discounting mechanism results in a delayed appreciation of the infant's true risk by about 4 weeks (Figure 2) and is due to the bundling of the three variables with resultant emphasis on lack of Stage and Plus. By indicating risk earlier, tROP‐SS also displays a notable characteristic downslope in the untreated cohort in the first 5 weeks of screening (Figure 4).

Typically, Zone, Stage and Plus are required to achieve a TW‐ROP designation, with AROP being a notable exception. By weighting Zone I, Stage 3 and Plus high, due to their centrality in the determination of TW‐ROP, risk is captured earlier, and the entire team and family are aware of the risk.

Our study has several limitations. Not all patients completed their screenings within the study timeframe; for some patients, their screening will extend into 2025, so we may underestimate the prevalence of high risk and TW‐ROP. Second, while tROP‐SS performed well across a more diverse set of infants, our study is limited in that it is retrospective and unmasked in nature. The tROP‐SS is being evaluated prospectively by 12 NICUs in the SUNDROP network, and the results will be reported when they become available.

We evaluate the performance of the mROP‐ActS relative to the tROP‐SS in infants at risk for ROP over a 7‐year period across 14 NICUs. The tROP‐SS was always able to both generate scores for each eye at each visit, demonstrate disease tempo relative to time showing the effect of worsening, giving neonatologists and parents timely information.

## Supporting information


Figure S1:

Table S1:
