# Supplementary material for: Multi‐site performance of the telemedicine retinopathy of prematurity severity score (tROP‐SS)
Source: Acta Ophthalmol. 2026 Feb 23;104(5):e590–5. doi: 10.1111/aos.70101 (PMC13353693; doi:10.1111/aos.70101)

**Supplemental Figure S1:** *(a) Individual trajectories of tROP-SS (red) and mROP-ActS (blue) for four sample patients. Each individual trajectory for each scoring system incorporates data that is pooled into the population estimate (b). Individual trajectories are then fed back into population estimates via partial pooling (c).*


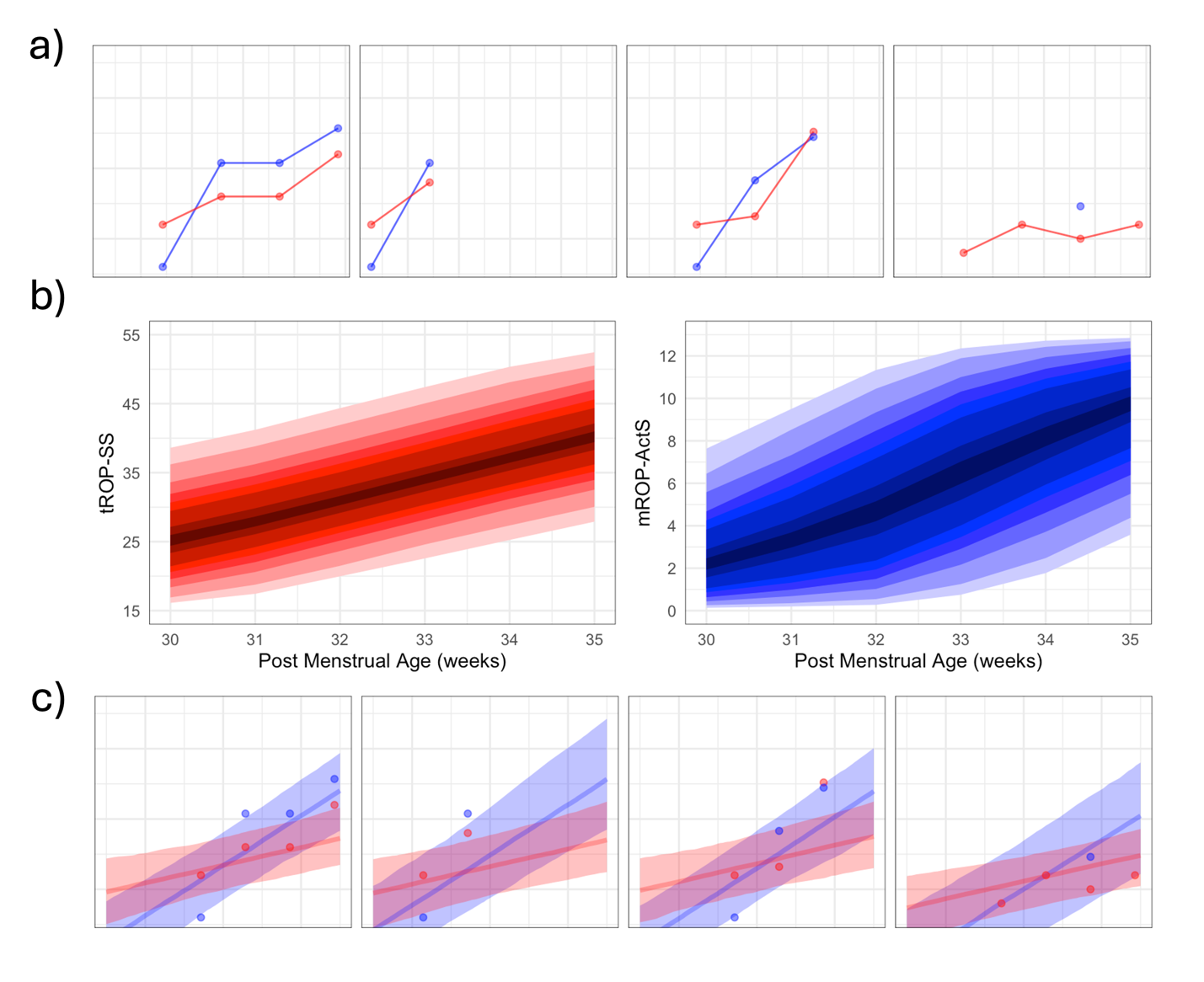

Supplement: Supplementary file 1 — Figure S1: [file AOS-104-e590-s002.docx]
