# Supplementary material for: Multi‐site performance of the telemedicine retinopathy of prematurity severity score (tROP‐SS)
Source: Acta Ophthalmol. 2026 Feb 23;104(5):e590–5. doi: 10.1111/aos.70101 (PMC13353693; doi:10.1111/aos.70101)
Supplement: Supplementary file 2 — Table S1: [file AOS-104-e590-s001.docx]

| Reason for invalid mROP-ActS | n (eyes) | % |
| --- | --- | --- |
| Only Posterior Zone II | 988 | 59.53% |
| Only Regression | 400 | 24.13% |
| Only Pre-plus | 214 | 12.92% |
| Posterior Zone II AND Pre-plus | 36 | 2.16% |
| Pre-plus AND Regression | 11 | 0.68% |
| Posterior Zone II AND Regression | 4 | 0.23% |
| Zone I, Incomplete Vascularization, AND Plus | 3 | 0.17% |
| Reactivation | 2 | 0.11% |
| Posterior Zone II, Pre-plus, AND Regression | 1 | 0.06% |
| Total Eyes Invalid | 1660 | 100% |

**Supplemental Table S1:** *Proportion of invalid mROP-ActS per eye categorized by various reasons*
